# Supplementary material for: Structural insights into the mechanism of pancreatic KATP channel regulation by nucleotides
Source: Nat Commun. 2022 May 19;13:2770. doi: 10.1038/s41467-022-30430-4 (PMC9120461; doi:10.1038/s41467-022-30430-4)
Supplement: Supplementary file 1 — Supplementary Information [file 41467_2022_30430_MOESM1_ESM.pdf]

Supplementary Figures 1-7 and Supplementary Table 1 related to:

Structural insights into the mechanism of pancreatic K_{ATP} channel regulation by nucleotides

Authors

Mengmeng Wang [1,2,3], Jing-Xiang Wu [1], Dian Ding [1,2,3], and Lei Chen [1,2,3*]

Affiliations

[1] State Key Laboratory of Membrane Biology, College of Future Technology, Institute of Molecular Medicine, Peking. University, Beijing Key Laboratory of Cardiometabolic Molecular Medicine, Beijing 100871, China.

[2] Peking-Tsinghua Center for Life Sciences, Peking University, Beijing 100871, China.

[3]Academy for Advanced Interdisciplinary Studies, Peking University, Beijing 100871, China.

*Corresponding author: Lei Chen,

Correspondence to: chenlei2016@pku.edu.cn

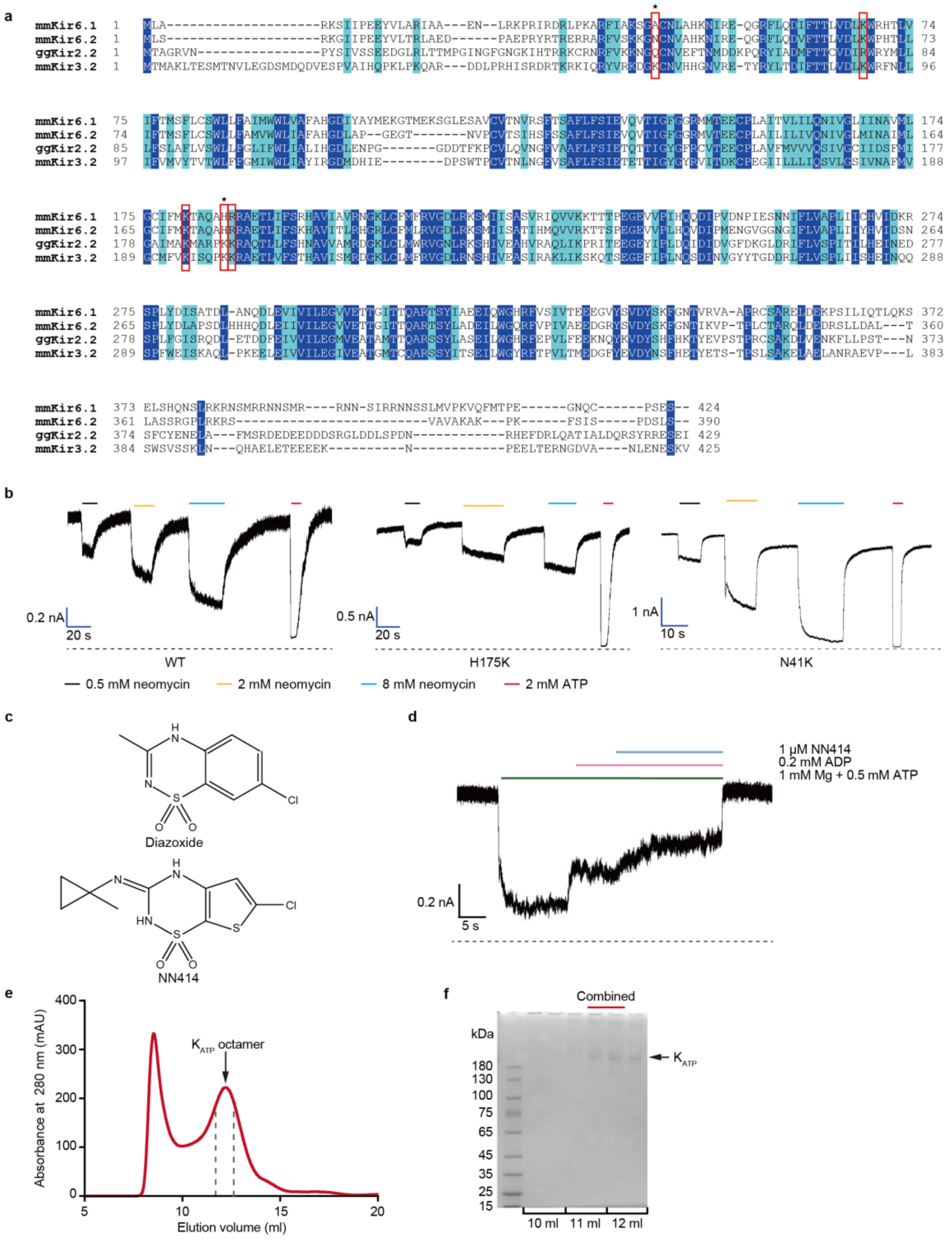

Supplementary Figure 1 | Characterization of K_{ATP} H175$K_{cryo-EM}$. a, Sequence alignment of Kir6.1 from *Mus musculus* (mmKir6.1), mmKir6.2, Kir2.2 from *Gallus gallus* (ggKir2.2), and mmKir3.2. Identical residues are in the blue background while residues with similarity more than 50% are in cyan background. Positively charged residues that might participate in PI(4,5)P_2 binding were boxed in red. N41 and H175 of mmKir6.2 were labeled by asterisks above. **b**, The inside-out recordings of neomycin inhibition of wild-type K_{ATP} channel and K_{ATP} channel with H175K or N41K mutation on Kir6.2. **c**, Chemical structures of NN414 and diazoxide show their similarities. **d**, Representative inside-out current of H175$K_{cryo-EM}$. **e**, Size-exclusion chromatography (SEC) elution profile of H175$K_{cryo-EM.}$ Fractions between the dashed lines were K_{ATP} octamer used for cryo-EM sample preparation. **f**, SDS-PAGE of H175$K_{cryo-EM}$ Fractions labeled by red bars were collected for cryo-EM sample preparation. 4 - 15% gradient gel was used and stained by coomassie brilliant blue R-250.

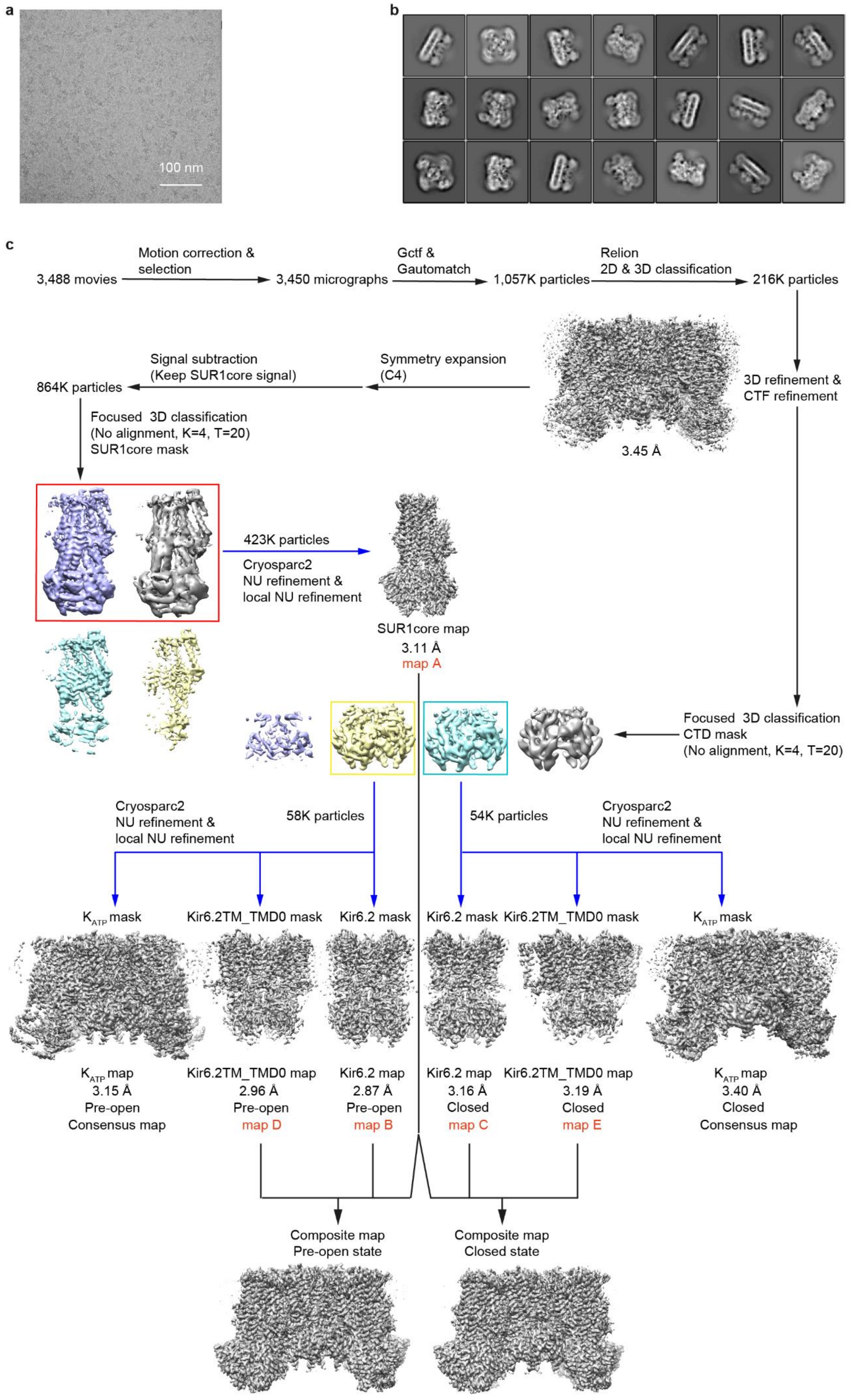

Supplementary Figure 2 | Cryo-EM image processing flow-chart. a, Representative raw micrograph. **b,** Representative 2D class averages of H175K$_{\text{cryo-EM}}$. **c,** Flow-chart of cryo-EM image processing for H175K$_{\text{cryo-EM}}$ dataset.

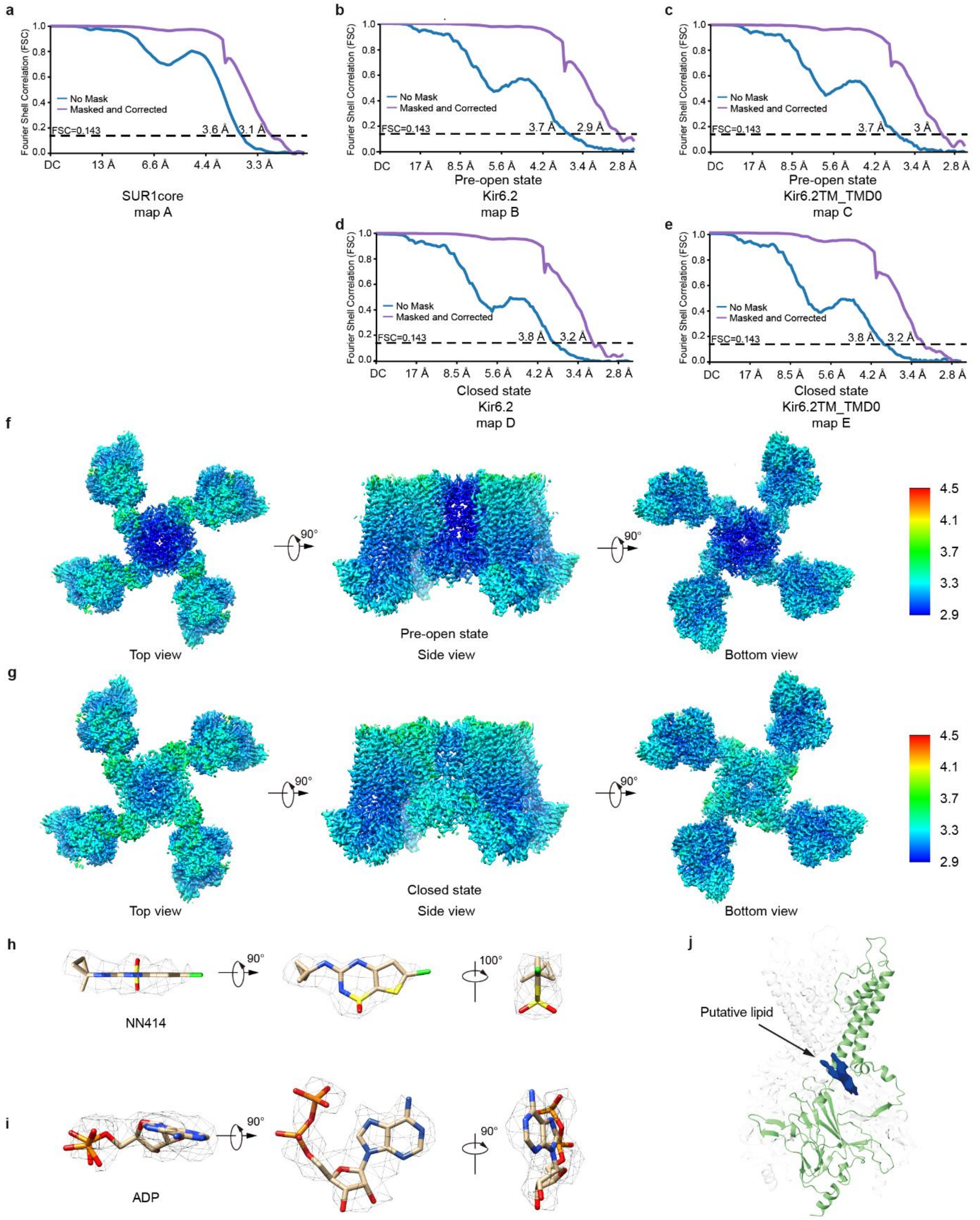

Supplementary Figure 3 | Electron densities of K$_{ATP}$ H175K$_{cryo\text{-}EM}$. a-e, FSC curves of each focus refined map shown in Fig. S2. **f-g**, Local resolution estimation of the composite maps. **h**, Electron density maps of NN414 viewed from different angles. NN414 is shown as sticks. Electron density is shown as gray mesh. **i**, Electron density maps of ADP bound in Kir6.2 viewed from different angles. **j**, Electron density of putative lipid bound in Kir6.2 is shown in blue.

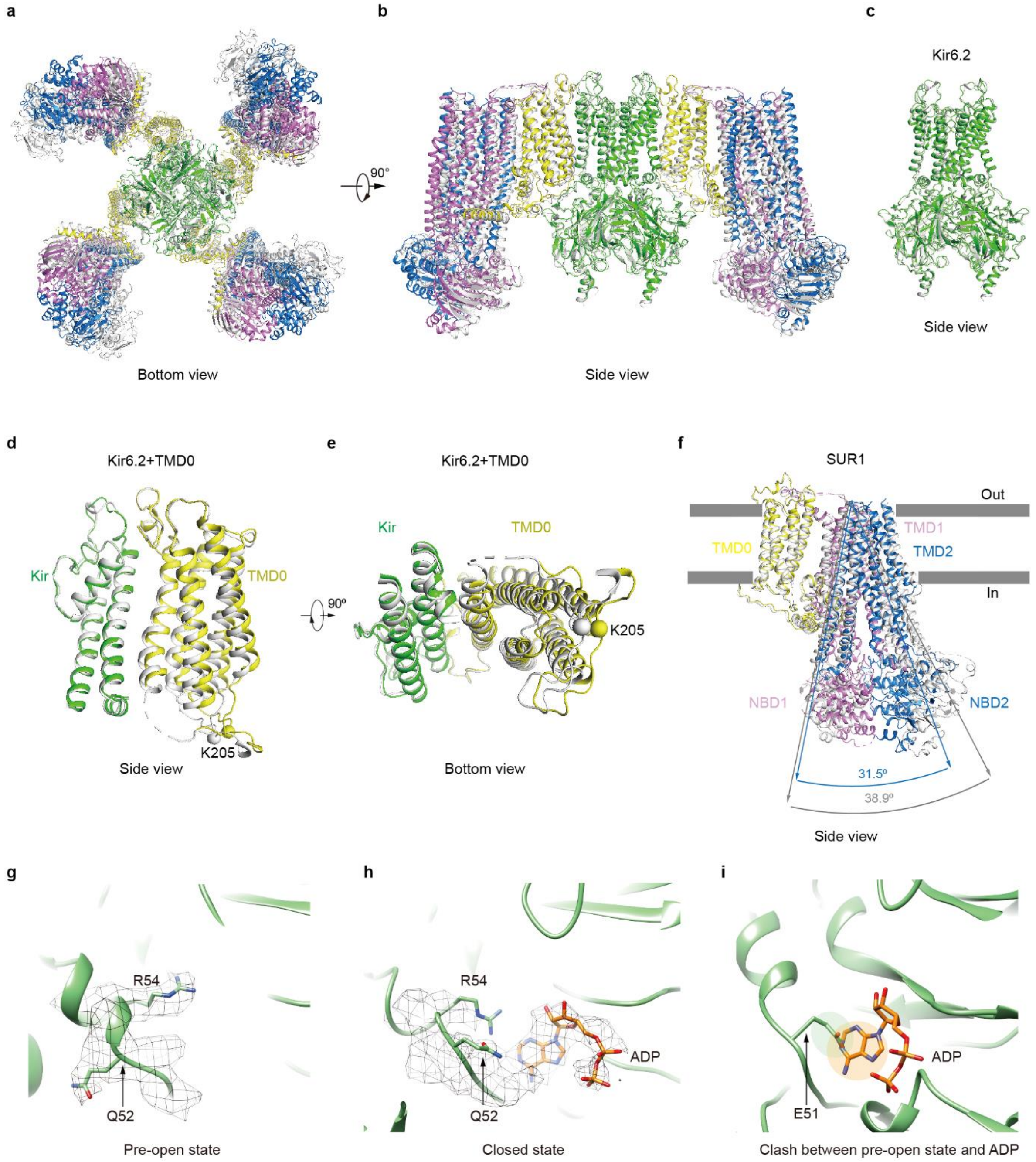

Supplementary Figure 4 | Structural comparison between the closed state of K_{ATP} H175$K_{cryo\text{-}EM}$ and K_{ATP} in the presence of ATP+RPG. a-b, Superposition of the closed state structure of H175$K_{cryo\text{-}EM}$ and K_{ATP} in the presence of ATP+RPG (PDB ID: 6JB1) by aligning the TMD of Kir6.2. The bottom view (**a**) and side view (**b**) are shown. H175$K_{cryo\text{-}EM}$ is colored according to **Fig. 1**. The structure of K_{ATP} in the ATP+RPG state is colored in gray. **c,** Structural comparisons of Kir6.2 in the closed state of H175$K_{cryo\text{-}EM}$ (green) and in the ATP+RPG state (gray). **d-e**, Structural comparisons of Kir6.2 and TMD0 in the closed state of H175$K_{cryo\text{-}EM}$ (colored) and in the ATP+RPG state (gray). The Cα positions of K205 are shown as spheres. **f,** Structural comparison of SUR1 in the closed state of H175$K_{cryo\text{-}EM}$ (colored) and in the ATP+RPG state (gray). Angles between M10 and M16 in the two structures are measured and labeled. **g,** The electron densities of regions around Q52 of Kir6.2 in pre-open state are shown as meshes. Side chains of Q52 and R54 are shown as sticks. **h,** The electron densities of regions around Q52 of Kir6.2 in closed state are shown as meshes. ATP and side chains of Q52 and R54 are shown as sticks. **i,** ADP (colored in orange) was modelled into the nucleotide binding site in the pre-open state. The obvious sterical clash between ADP and the side chain of E51 is denoted.

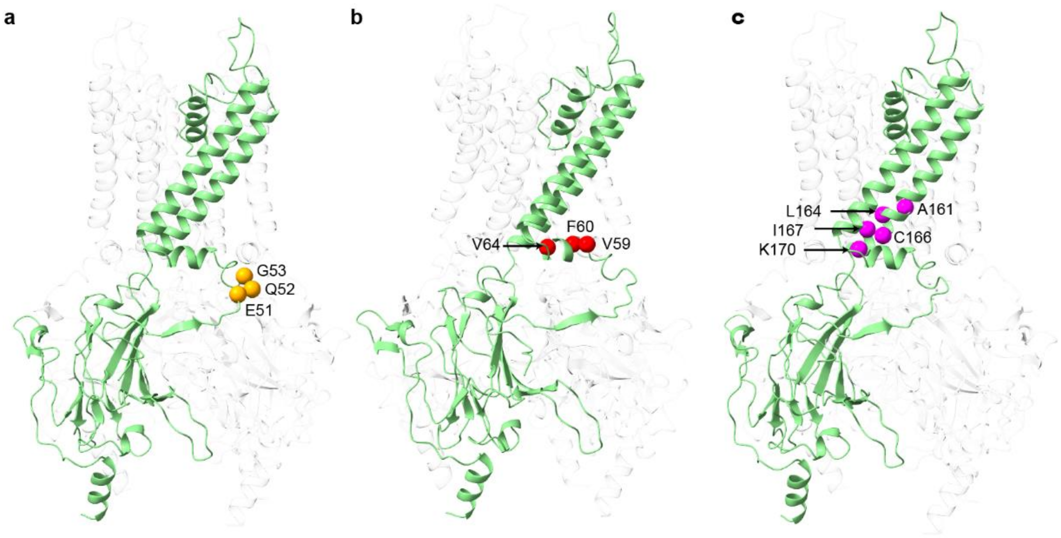

Supplementary Figure 5 | The spatial distribution of neonatal diabetes mutations mapped onto the structure of Kir6.2. Only mutations outside the ATP binding pocket of Kir6.2 are shown. **a,** Mutations on the βA-IH linker. Their Cα positions were shown as orange spheres. **b,** Mutations on IH helix (red spheres). **c**, Mutations on M2 helix (magenta spheres).

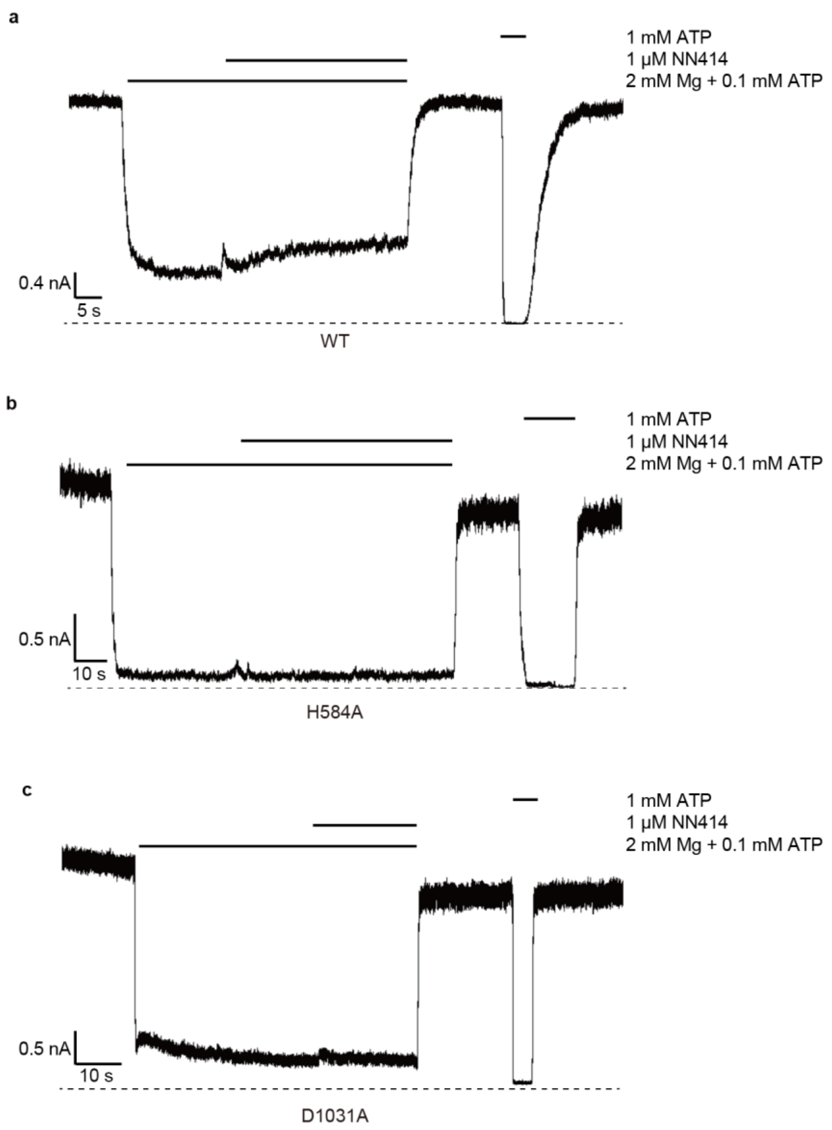

Supplementary Figure 6 | The activation of K_{ATP} channel currents by NN414. a, Inside-out recording of 1 µM NN414 activation of wild type K_{ATP} channel in the presence of 2 mM Mg^{2+} and 0.1 mM ATP. **b,** NN414 activation of H584A K_{ATP} channel mutant. **c,** NN414 activation of D1031A K_{ATP} channel mutant.

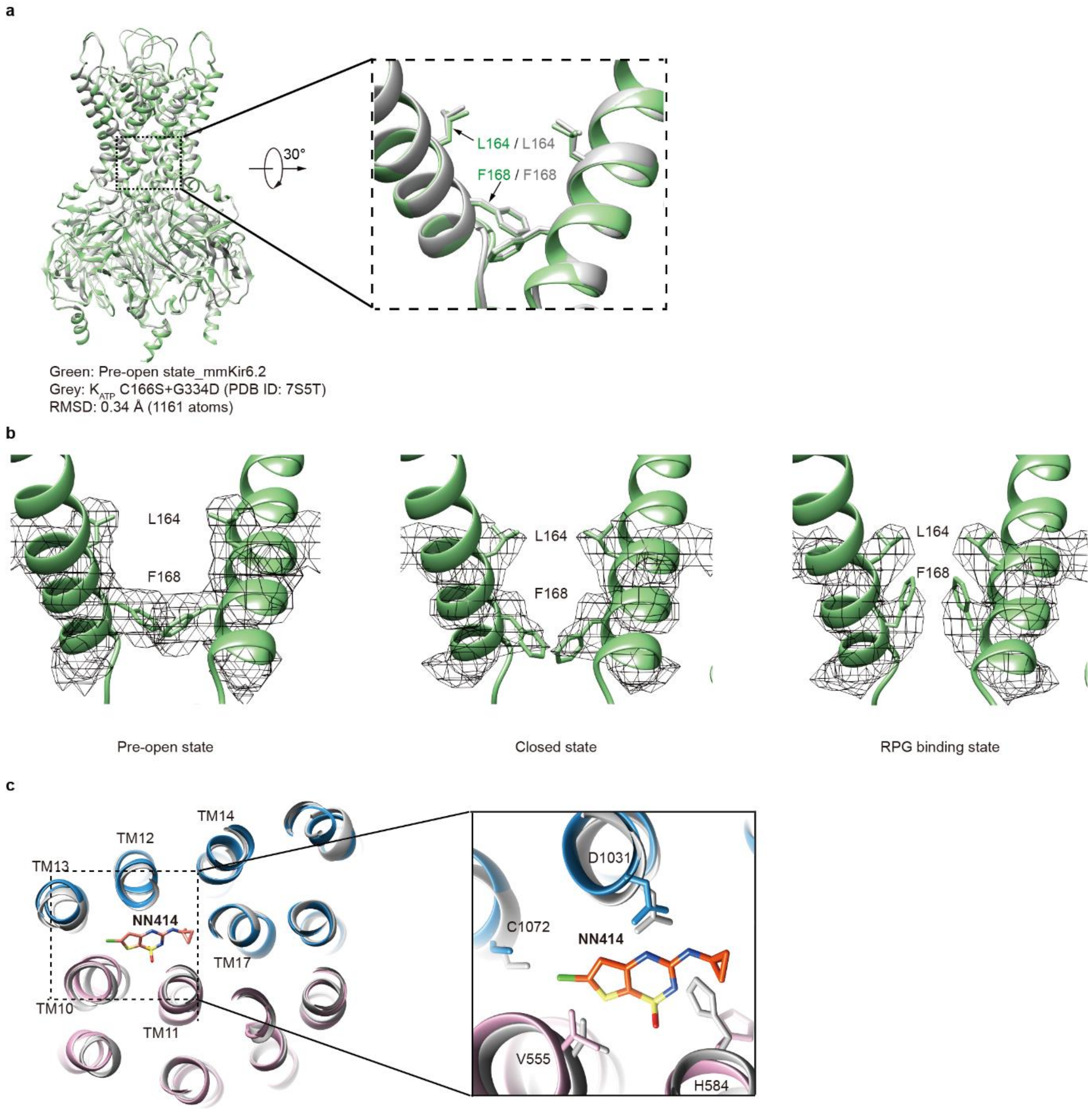

Supplementary Figure 7 | Structural comparison between the pre-open state of K_{ATP} H175K$_{cryo\text{-}EM}$ and K_{ATP} C166S+G334D$_{cryo\text{-}EM}$. a, Structural comparison of Kir6.2 between the pre-open state of K_{ATP} H175K$_{cryo\text{-}EM}$ (in green) and K_{ATP} C166S+G334D$_{cryo\text{-}EM}$ (in gray, PDB ID: 7S5T). The RMSD value is 0.34 Å. **b,** Electron density of L164 and F168 in the pre-open state, closed state and RPG binding state (PDB ID: 6JB1). **c,** Structural comparison of SUR1 between K_{ATP} H175K$_{cryo\text{-}EM}$ (colored in purple and blue) and K_{ATP} C166S+G334D$_{cryo\text{-}EM}$ (colored in grey). NN414 binding induces the enlargement of its binding site surrounded by TM10, TM11, TM12, TM14, and TM17. The side chains of V555, H584, C1072, and D1031 are shown as sticks to denote the conformational changes of SUR1 induced by NN414 binding.

Supplementary Table 1
Cryo-EM data collection, refinement and validation statistics

$H175K_{cryoEM}$ sample<br>PDB ID<br>EMDB ID	Pre-open state<br>7W4O<br>EMD-32310	Closed state<br>7W4P<br>EMD-32311
**Data collection and processing**		
Magnification	105,000 ×	
Voltage (kV)	300	
Electron exposure (e^-/Å^2)	50	
Defocus range (μm)	-1.5 to -1.8	
Pixel size (Å)	1.324	
Symmetry imposed	*C4*	
Initial particle images (no.)	1,057,599	
Final particle images (no.)	58,955/ 58,955/ 423,785*	54,546/ 54,546/ 423,785*
Map resolution (Å)	2.87/ 2.96/ 3.11*	3.16/ 3.19/ 3.11*
FSC threshold	0.143	
Map resolution range (Å)	2.72 - 250	
**Refinement**		
Initial model used (PDB code)	6JB1, 5YWC	
Model resolution (Å)	3.15	3.28
FSC threshold	0.5	0.5
Model resolution range (Å)	3.15 - 250	3.28 - 250
Map sharpening B factor (Å^2)	-104.6/ -104.4/ -135.3*	-111.4/ -114.2/ -135.3*
Model composition		
Non-hydrogen atoms	54,748	54,408
Protein residues	6944	6,904
Ligands	20	24
B factors (Å^2)		
Protein	59.38	46.87
Ligand	61.79	58.61
R.m.s. deviations		
Bond lengths (Å)	0.005	0.006
Bond angles (°)	1.062	1.089
Validation		
MolProbity score	1.64	1.61
Clashscore	5.00	4.61
Poor rotamers (%)	2.09	2.04
Ramachandran plot		
Favored (%)	97.26	97.24
Allowed (%)	2.62	2.76
Disallowed (%)	0.12	0.00

*Numbers indicate the resolutions of focus refined maps for Kir6.2/ Kir6.2TMD+SUR1TMD0 /SUR1 ABC transporter module, respectively.
