# Supplementary material for: Structural insights into the mechanism of pancreatic KATP channel regulation by nucleotides
Source: Nat Commun. 2022 May 19;13:2770. doi: 10.1038/s41467-022-30430-4 (PMC9120461; doi:10.1038/s41467-022-30430-4)
Supplement: Supplementary file 2 — Description of Additional Supplementary Files [file 41467_2022_30430_MOESM2_ESM.pdf]

File name: Supplementary Movie 1

Description: Conformational changes of K_{ATP} channel from the close state to the pre-open state.

File name: Supplementary Movie 2

Description: Conformational changes of SUR1 from the repaglinidebound state to the Mgnucleotides and NN414-bound state.
